# Bactericidal and In Vitro Cytotoxicity of *Moringa oleifera* Seed Extract and Its Elemental Analysis Using Laser-Induced Breakdown Spectroscopy

**DOI:** 10.3390/ph13080193

**Published:** 2020-08-13

**Authors:** Reem K. Aldakheel, Suriya Rehman, Munirah A. Almessiere, Firdos A. Khan, Mohammed A. Gondal, Ahmed Mostafa, Abdulhadi Baykal

**Affiliations:** 1 Department of Biophysics, Institute for Research & Medical Consultations (IRMC), Imam Abdulrahman Bin Faisal University, Dammam 31441, Saudi Arabia; reem.k.dakheel@gmail.com (R.K.A.); malmessiere@iau.edu.sa (M.A.A.); 2 Department of Physics, College of Science, Imam Abdulrahman Bin Faisal University, Dammam 31441, Saudi Arabia; 3 Department of Epidemic Diseases Research, Institute for Research & Medical Consultations (IRMC), Imam Abdulrahman Bin Faisal University, Dammam 31441, Saudi Arabia; 4 Department of Stem Cell Research, Institute for Research & Medical Consultations (IRMC), Imam Abdulrahman Bin Faisal University, Dammam 31441, Saudi Arabia; fakhan@iau.edu.sa; 5 Department of Physics, Laser Research Group, King Fahd University of Petroleum & Minerals, Box 372, Dhahran 31261, Saudi Arabia; 6 Department of Pharmaceutical Chemistry, College of Clinical Pharmacy, Imam Abdulrahman Bin Faisal University, Dammam 31441, Saudi Arabia; ammostafa@iau.edu.sa; 7 Department of Nanomedicine Research, Institute for Research & Medical Consultations (IRMC), Imam Abdulrahman Bin Faisal University, Dammam 31441, Saudi Arabia; abaykal@iau.edu.sa

**Keywords:** antibacterial, anticancer, GC-MS, LIBS, *Moringa oleifera*, seed extract

## Abstract

In the current study, we present the correlation between the capability of laser-induced breakdown spectroscopy (LIBS) to monitor the elemental compositions of plants and their biological effects. The selected plant, *Moringa oleifera*, is known to harbor various minerals and vitamins useful for human health and is a potential source for pharmaceutical interventions. From this standpoint, we assessed the antibacterial and in vitro cytotoxicity of the bioactive components present in *Moringa oleifera* seed (MOS) extract. Detailed elemental analyses of pellets of MOSs were performed via LIBS. Furthermore, the LIBS outcome was validated using gas chromatography–mass spectrometry (GC-MS). The LIBS signal was recorded, and the presence of the essential elements (Na, Ca, Se, K, Mg, Zn, P, S, Fe and Mn) in the MOSs were examined. The bactericidal efficacy of the alcoholic MOS extract was examined against *Escherichia coli (E. coli)* and *Staphylococcus aureus* *(S. aureus)* by agar well diffusion (AWD) assays and scanning electron microscopy (SEM), which depicted greater inhibition against Gram-positive bacteria. The validity and DNA nuclear morphology of human colorectal carcinoma cells (HCT-116) cells were evaluated via an MTT assay and DAPI staining. The MTT assay results manifested a profoundly inhibitory action of MOS extract on HCT116 cell growth. Additionally, MOS extracts produced inhibitory action in colon cancer cells (HCT-116), whereas no inhibitory action was seen using the same concentrations of MOS extract on HEK-293 cells (non-cancerous cells), suggesting that MOS extracts could be non-cytotoxic to normal cells. The antibacterial and anticancer potency of these MOS extracts could be due to the presence of various bioactive chemical complexes, such as ethyl ester and D-allose and hexadecenoic, oleic and palmitic acids, making them an ideal candidate for pharmaceutical research and applications.

## 1. Introduction

Traditional herbalists throughout the globe have emphasized the role of plants used as remedies to treat different diseases, like inflammation and bacterial infections [1]. Enormous research is being carried out, where plants are being tested for active compounds that have antibacterial, antifungal and anticancer activities [2,3]. One such plant is *Moringa oleifera* Lam. (MOL), which originates mainly from Africa, Asia and South America [4,5]. The moringa leaves and fruits are considered as vegetables in the Philippines, Thailand, India and Pakistan [6]. Recently, sundry countries (Mexico, Caribbean islands, Hawaii and Cambodia) have begun planting it for its plentiful health benefits and nutritional value in addition to its medicinal importance [7,8]. *Moringa* fruits and leaves have various biological applications in addition to being enriched with vitamins, minerals, and proteins. The WHO recommends using MOL as a food because of its superior nutritional values for human health [6]. It is a well-known fact that *Moringa* plants (leaves and fruits) were grown as for skin sanitation, an energy source and also to relieve tension [8,9]. Innumerable reports have discovered that MOL has numerous significant merits, such as antioxidant, antimicrobial, anticancer, anti-inflammatory, antiulcer, antihypertensive, anti-urolithic, anti-asthmatic, antidiabetic, analgesic, anti-aging, diuretic, cardiovascular, hepatoprotective, hypoglycemic and immunomodulatory characteristics [10,11,12,13]. The hypotensive, antibacterial and anticancer efficacies of the MOL leaves and fruits are due to the presence of sundry distinctive chemical compounds (benzyl glucosinolate, niazimicin, benzyl iso-thiocyanate complexes and pterygospermin) in their structure [11]. Several methods have been improved to extract the initial contents from MOL plants for the production of food supplements and medicines (with natural organic components) and the determination of their other health benefits. Extraction procedures based on pressurized liquid, ultrasound, microwaves and supercritical fluid have been introduced [14,15,16,17].

Nevertheless, all extraction mechanisms experience several restrictions in terms of using a large amount of harmful organic solvents, sample production proceedings and cost [13]. To get better results, LIBS has emerged as an efficient approach for the identification and quantification of elemental compositions from different medicinal plant extractions. Over the past decade, the LIBS method has broadly been exploited for analyzing chemical elements existing in diverse kinds of specimens. This analytical method has distinguishing properties, like cost effectiveness, real-time measurement, sensitivity, rapidity and in situ elemental analysis [18,19,20]. Additionally, this type of analysis is easy, eco-friendly and is less complicated when preparing the sample. It is known that intensive sample treatments frequently produce erroneous results due to the presence of contaminants and loss-related effects. Thus, the LIBS technique can accurately disclose the identity of various medicinal plants in a scientific manner. In addition, most edible plants consist of proteins, vitamins and minerals, which possess immense benefits to health [21,22].

The purpose of the study is to analyze the presence of different elements in *Moringa oleifera* seed (MOS) extracts by using the LIBS approach and to examine the biological activities of MOS extracts by evaluating the antibacterial and anticancer potencies.

## 2. Results

### 2.1. Qualitative Analysis of MOS Using LIBS

**Figure 1:**
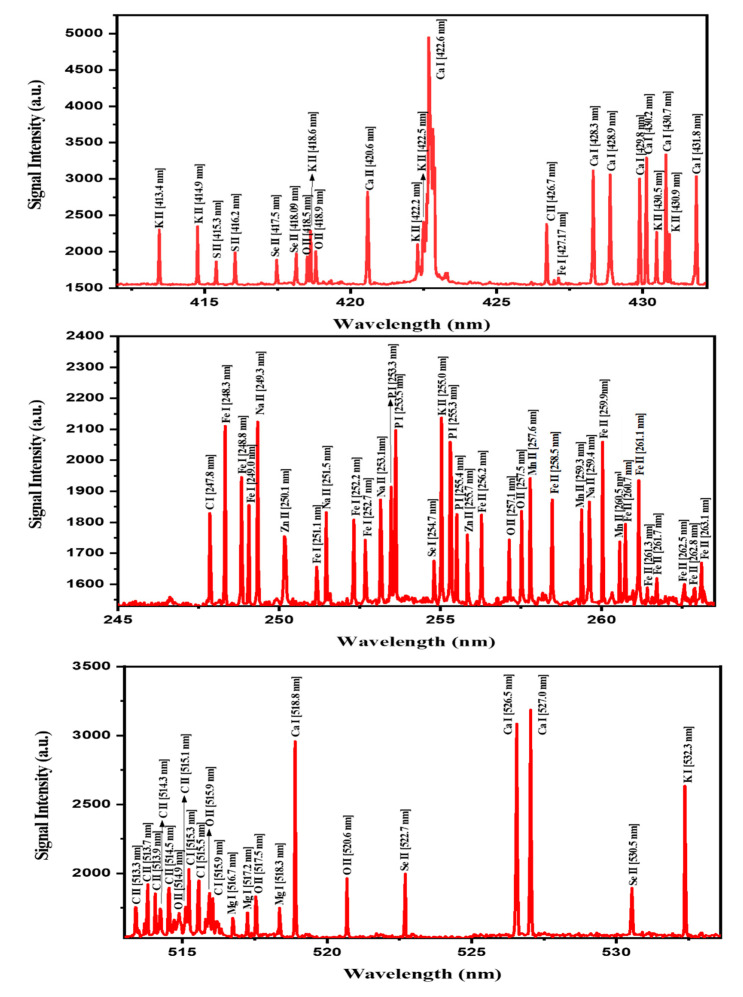
LIBS emission line spectra of various elements recorded in the wavelength range of 245–537 nm of MOS. The signature lines for different vital minerals present in MOSs are indicated in the figure.

The laser-induced breakdown spectroscopy (LIBS) spectra (Figure 1) of the MOS extract were recorded in the range of 200 to 800 nm. For the detection of the major elements, the spectra were recorded from different spots of the pellets and by scanning at a 50 nm wavelength range each time. In order to reduce the background noise and to enhance the LIBS signal intensity, LIBS parameters, including the delay times (delay between the incident laser pulse and the recording of spectra), the number of accumulations and laser energies, were optimized prior to the application of the LIBS setup for MOS sample analysis. The recorded LIBS spectra of the MOSs comprised various significant spectral peaks of the atomic and ionic lines (with varying intensities) related to the abundance of elements present in the tested MOS samples. Based on the NIST database, the recorded spectral lines were identified and classified in terms of the characteristic elements present in the MOSs, suggesting their significant role towards antibacterial and anticancer activities. Clearly, the measured LIBS spectra (Figure 1) exhibited the presence of vital elements such as Ca, K, Mg, P, S, Fe, Mn, Zn, Na and Se in MOS samples.

Table 1 presents the LIBS signal intensities of the spectral transition lines corresponding to various detected elements in MOSs. In accordance with the Boltzmann distribution, the intensities of the LIBS spectral lines have a direct relationship with the elemental contents (concentration) present in MOSs [23,24]. This correlation was attained by considering the intensity ratio of the detected elemental lines with that of the C line taken as the reference (247.8 nm). The achieved intensity ratios of the elements Ca, K, Mg, P, S, Fe, Mn, Zn, Na and Se were in the ranges of 2.7–1.7, 1.2–1.4, 0.9, 1.1, 1.1, 1.1–0.9, 1.0, 0.9, 1.2 and 1.1, respectively, which were consistent with those reported in the literature [16].

### 2.2. Volatile Content Analyses of MOS using GC-MS

**Figure 2:**
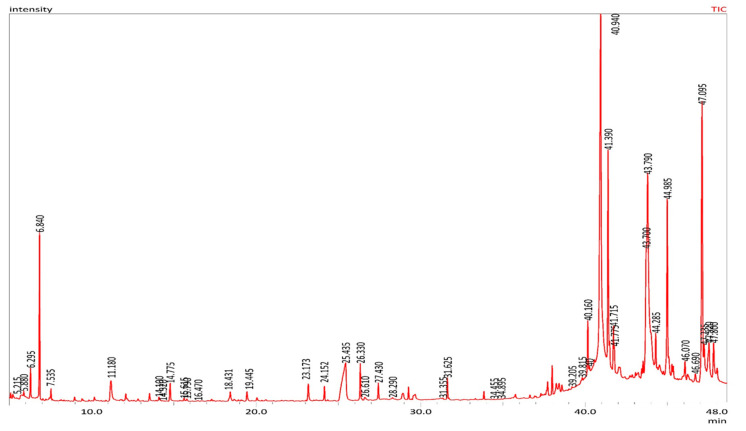
GC-MS chromatograph of MOS.

The GC-MS analyses of the *Moringa oleifera* seed (MOS) showed the presence of 114 volatile complexes with diverse chemical groups: fatty acids, esters, ketones, alcohols, aldehydes, and hydrocarbons. Table 2 shows the retention times and Figure 2 displays the percentage composition of all the identified chemical compounds in MOS. The main types were oleic acid (22.53%), 2-3-di-hydroxy-propyl (13.48%), 9-octa-decenoic acid (*Z*), 2-3-di-hydroxy-propyl ester (11.35%), docosenamide (6.04%), ethyl oleate (6.03%), 1-3-propanediol, 2-ethyl-2-(hydroxyl-methyl) (5.52%), oleic anhydride (3.96%), 2-propanone and 1-1-dimethoxy (3.86%). These MOSs contain fatty acids and their ester derivatives (65.45%), alcohols (9.4%), nitrogen compounds (9.09%), ketones (5.34%) and aldehydes (2.88%). To form esters, fatty acids and alcohols in plants may undergo esterification.

### 2.3. *In vitro* Cytotoxic Activity of MOS

**Figure 3:**
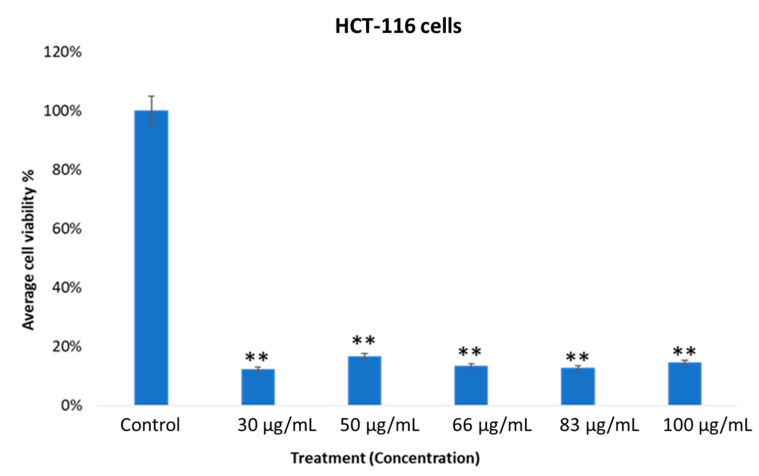
Effect of MOS extract on colon cancer cells (HCT-116) after treatment for 48 h with different concentrations. The average cell viability was calculated by MTT assay (** *p* < 0.01).

The cell viability of the MOS extract-treated HCT-116 cells was evaluated using an MTT assay after 48 h. The percentage of cell viability of the MOS extract-treated HEK-293 and HCT-116 cells was determined. The MTT assay examined the percentage of cell viability. The number of viable cells present in the control group was compared with the MOS-treated cells. In Figure 3, we show that cancer cells (control group, without MOS treatment) showed 100% cell viability, whereas cancer cells treated with MOS extract showed a significant decrease, which suggests that MOS extract could induce a significant drop in the viability of cancerous cells compared to control ones (without treatment using the MOS extract).

**Figure 4:**
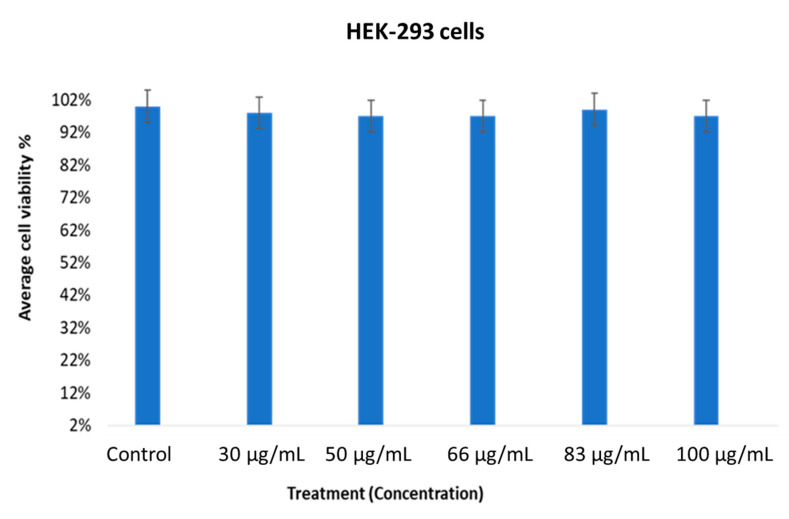
Effect of MOS extract on normal cells (HEK-293) after treatment for 48 h treated different concentrations. The average cell viability was calculated by MTT assay.

The average viability of the MOS extract-treated cancer cells at various concentrations showed quite encouraging outcomes, with *p* < 0.01. The specificity of the MOS extract in the concentration range of 30 to 100 µg/mL on normal cells was inspected using MTT assays after treatment for 48 h (Figure 4). When MOS extract was tested on normal cells (HEK-293), we found no inhibitory action.

The MOS extract showed content-dependent specificity when the data were taken from three replications. A Student’s *t*-test was used to understand the difference between the two treated groups and the results are presented as the mean ± standard deviation (SD).

### 2.4. Nuclear Breakdown of MOS Extract-Treated Cancerous Cells

**Figure 5:**
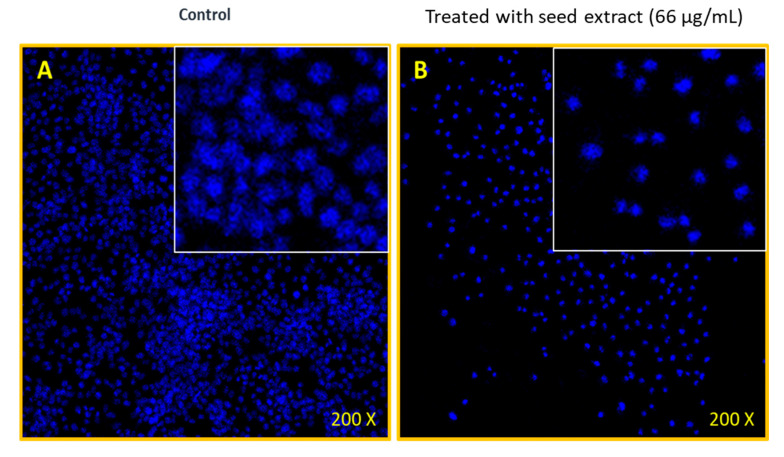
Confocal staining by DAPI. (**A**) The HCT-116 cells (non-treated) and (**B**) HCT-116 cells treated with MOS (66 µg/mL), 200× magnifications.

Figure 5 illustrates the MOS extract-treated and untreated cell morphology that was imaged using confocal scanning microscopy (CSM). The MOS extract-treated cancer cells exhibited stronger inhibitory action (Figure 5A) than the control sample (Figure 5B). The CMS images of the nuclear cell morphology of both control (untreated) and MOS extract-treated (66 µg/mL) samples after stained by DAPI showed a substantial loss (nuclear disintegration) because of the treatments.

It was deduced that the MOS extract has strong anti-cancerous activities in colon cancer cells (as supported by the GC-MS analysis).

### 2.5. Antibacterial Efficacy of MOS Extract

The antibacterial potency of the MOS extract was assessed on Gram-negative and Gram-positive bacteria using the AWD method, wherein the inhibited areas due to antibacterial action around the inoculated wells were measured. This zone of inhibition was produced by the diffusion of the active chemical constituents present in the MOS extract. These results confirm the impact of the MOS extract on *S. aureus* and *E. coli*. Interestingly, the MOS extract was found to produce better antibacterial action in *S. aureus* bacteria than *E. coli*.

**Figure 6:**
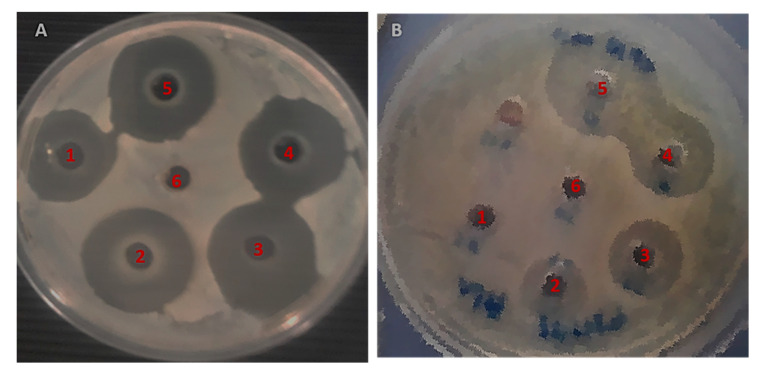
Agar well diffusion (AWD) plates showing the inhibition zones. (**A**) *S. aureus* and (**B**) *E. coli.* **1**: 50 µg/mL, **2**: 100 µg/mL, **3**: 150 µg/mL, **4**: 200 µg/mL, **5**: 250 µg/mL of MOS, **6**: Control (DH_2_O).

**Figure 7:**
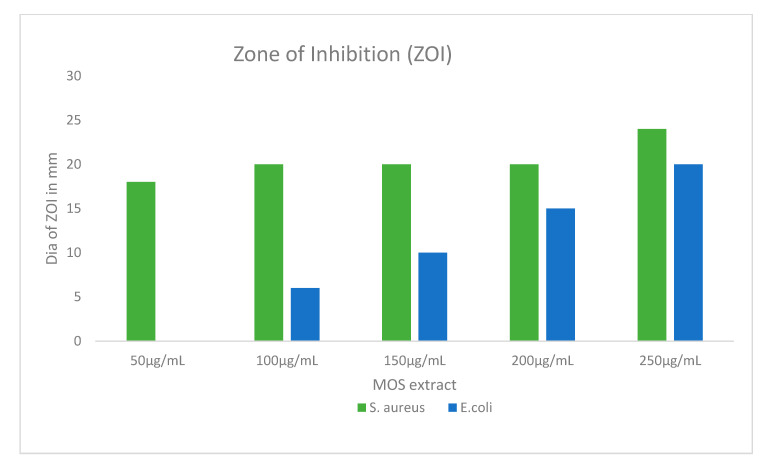
The graphical illustration of the zones of inhibition in millimeters (mm) examined in both *S. aureus* and *E. coli* after treatment using different concentrations of MOS extract. Data are the means ± SD of three different experiments.

In Figure 6, the MOS extract shows concentration-dependent inhibition of *S. aureus* bacteria, with inhibition zones ranging from 18 to 24 mm. On the other hand, the inhibition zones of *E. coli* were found to range from 6 to 20 mm. The maximum and minimum inhibition zone diameters were acquired with the corresponding extract contents of 250 and 50 µg/mL (Figure 6 and Figure 7). No inhibition was observed in *E.coli* with 50 µg/mL, whereas a higher concentration showed inhibition of the bacteria.

**Figure 8:**
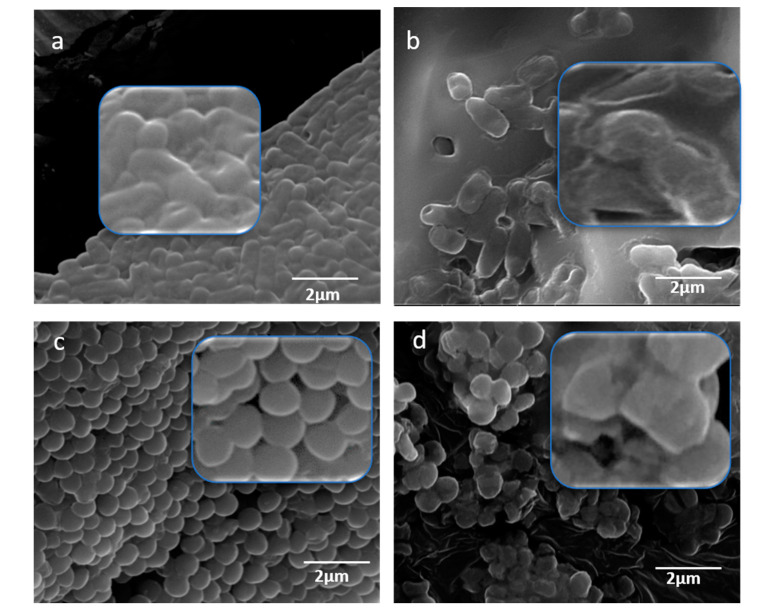
SEM micrographs for the study of morphogenesis by MOS extract. (**a**) *E. coli* cells (non-treated) and (**b**) treated *E. coli* cells, (**c**) *S. aureus* cells (non-treated) and (**d**) treated *S. aureus* cells.

The morphological changes in bacteria treated with MOS were studied using SEM. The untreated cells of *E. coli* were seen as regular rod-shaped cells with smooth cell surfaces (Figure 8a). The *E. coli* subjected to treatment with MOS appeared as damaged cells (Figure 8b) and the cell number also showed a reduction with a significant alteration of the cell wall and membrane. Similarly, the treatment of *S. aureus* with MOS extract also showed significant morphological changes in the structure and number of cells (Figure 8d), whereas the control *S. aureus* cells appeared as regular cocci with intact cell surfaces (Figure 8c). The damaged cells lost their cellular integrity, which led to the death of bacterial cells.

## 3. Discussion

The LIBS and GC-MS techniques were utilized for the identification and quantitation of the elements existing in the MOS extract. For the first time, the anticancer and antibacterial activities of the MOS extract were assessed. Our results show the presence of diverse elements in MOSs, as confirmed by different methods. The GC-MS results of the MOS extract verified the existence of diverse anticancer and antimicrobial compounds. The spectra from different points on the surface of MOS pellets were examined by the LIBS method. The outcomes proved that MOSs are rich in different minerals that are useful to humans as food and medicine. Besides, the seeds have an influential role in antibacterial activity because of the availability of the following elements: K, Fe, P, Ca, Mg, S, Mn, Na, Se and Zn. It is clear that the amounts of Ca, K and Mg present in the MOSs were greater than the other elements. These results give evidence that MOSs are rich in different minerals, which are highly useful to humans as food supplements and medicine, indicating their remarkable impact in regulating the level of blood pressure, blood lipids, regulating the stomach function, protecting the liver, strengthening the bones, generating protein and enhancing the immunity of the human body [23,24,25]. Furthermore, the existence of Se in MOSs plays a vital role by protecting from fatal diseases like cancer, cardiovascular disease, cognitive decline, and thyroid disease. Moreover, these seeds exhibit a powerful antibacterial activity due to the presence of the detected elements, which is evidenced clearly by the antimicrobial studies conducted in this work.

The analysis of GC-MS of the MOSs showed the presence of 114 volatile complexes with diverse chemical groups: fatty acids, esters, ketones, alcohols, aldehydes, and hydrocarbons. To form esters, fatty acids and alcohols in plants may undergo esterification [19]. It has been reported that most of these compounds possess anticancer activities. In addition, palmitic acid shows selective cytotoxicity against human leukemic cells. The fatty acids also possess both antifungal and antibacterial activities. The omega-9 fatty acid was a detected primary complex (oleic acid) in the MOSs, which has numerous human health benefits (preventing ulcerative colitis and reducing blood pressure with remarkable antioxidant efficiency [26,27]). Therefore, GC-MS measurement proved the presence of numerous fatty acids and their related esters (cis-9-hexadecenoic acid (palmitoleic), oleic acid, octadecanoic (stearic) acids, n-hexadecenoic acid) and alcohols. It was reported that most of these compounds have anticancer activity. For example, D-allose inhibits cancer cell growth at G1 phase [17]. Palmitic acid has selective cytotoxicity against leukemic cells in humans. Fatty acids also have antifungal and antibacterial effects [28].

In the present study, the biological activities of MOS were evaluated. The cell viability of the MOS extract-treated (for 48 h) HCT-116 (cancer) cells was assessed via MTT assay. The viability status of the extract-treated HEK-293 (normal) and HCT-116 cells was determined. It was inferred that the MOS extract has strong anticancer activity in colon cancer cells. However, few reports done on MOS extract have shown that the enhanced anticancer activity of the extract correlated with the occurrence of high oleic acid and fatty acid contents [21]. Previously, it has been shown that *Moringa* plants (leaves and fruits) have been used for various applications, such as antioxidant, antimicrobial, anticancer, anti-inflammatory, antiulcer, antihypertensive, anti-urolithic, antidiabetic, anti-asthmatic, anti-aging, analgesic, diuretic, cardiovascular, hepatoprotective, hypoglycemic and immunomodulatory uses [22]. As per our knowledge, there is no information on whether *Moringa* leaf extract or seed extract cause any differential response in cancer cells. Nevertheless, it would be interesting to do a comparative study where the effects of *Moringa* seed and *Moringa* leaf extracts are examined in cancer cells. We have used HEK-293 (human embryonic kidney) cells as normal cells to compare the MOS anticancer activity to human colon cancer cell line HCT-116. The purpose was to check whether MOS extracts produce any cytotoxic effects in normal cells or not. In our studies, we have found that MOS extracts produce inhibitory action in colon cancer cells (HCT-116), whereas no inhibitory action was found using the same concentrations of MOS in HEK-293 cells (non-cancerous cells), which suggests that MOS extracts could be non-cytotoxic to normal cells.

MOS has 114 volatile complexes with diverse chemical groups: fatty acids, esters, ketones, alcohols, aldehydes and hydrocarbons. The hypotensive, antibacterial and anticancer efficacies of the MO leaves and fruits are due to presence of sundry distinctive chemical compounds (benzyl glucosinolate, niazimicin, benzyl iso-thiocyanate complexes and pterygospermin) in their structure [6]. Several methods have been improved to extract the initial contents from MOL plants for the production of food supplements medicines (with natural organic components) and the determination of their other health benefits. While we do not know the molecular mechanism of the anticancer activities of MOS extract in cancer cells, the role of the caspase signaling pathway cannot be ruled out in the process of programmed cell death. It would be interesting to study the different caspases, such as caspase-3 and caspase-9 in MOS extract-induced cell death.

The antimicrobial potency of the MOS extract was assessed in the Gram-negative and Gram-positive bacteria via AWD, wherein the inhibited areas around the inoculated wells were measured. Due to the occurrence of varying cell components, there is a discrepancy in the bactericidal action of the MOS extract for the two different types of test bacteria [29].

The study of morphogenesis using SEM showed that MOS extract affects the cell wall at the initial stage, and later penetrates and accumulates at the surface membrane. This leads to an interruption in the metabolic activities of bacterial cells and initiates cell death [30]. The present study illustrates that the MOS extract causes damage to the microbial cell surface, thereby causing significant antibacterial activity. The penetration of the extract into the bacterial cell alters the membrane integrity by structural alterations, the loss of membrane proteins, etc.

Natural compounds like fatty acids, esters, ketones, alcohols, aldehydes and hydrocarbons have been reported in a variety of plants [31]. The mechanisms of the antibacterial activities of such compounds is linked to their high affinity towards lipids due to their hydrophobic characteristics. Their antibacterial actions are evidently related to this lipophilic nature and to the bacterial membrane structure [32]. This leads the compounds to penetrate the cellular membrane of the microbial cell, which enhances the fluidity and permeability of membrane, alters the topology of membrane proteins and inflicts disruption in the respiration chain [33,34].

The present study indicated the presence of phenolic compounds and these are reported to disrupt the cell membrane, leading to the inhibition of cell metabolism causing the leakage of cellular content [35]. It has been found that phenolics inhibit processes associated with the cell membrane, for example, electron transport, ions, protein translocation, phosphorylation and other enzyme-dependent reactions. Therefore, the disturbed permeability of the cytoplasmic membrane may lead to cell death [36]. The interaction of plant bioactive compounds with bacterial cell membranes results in the inhibition of several Gram-positive and Gram-negative bacteria [37]. It has also been indicated that Gram-positive bacteria are more susceptible to the antibacterial action of natural compounds like fatty acids, esters, ketones, alcohols, aldehydes and hydrocarbons, compared to Gram-negative bacteria [38]. This is also concordant with the current study, owing to the fact that Gram-negative bacteria have an outer layer surrounding their cell wall, limiting the access of hydrophobic compounds.

Therefore, MOSs can serve as an antibacterial component that can be further studied and recommended as a potential antimicrobial and anticancer therapy. This natural source can be further evaluated and upgraded for pharmaceutical application.

## 4. Materials and Procedures

### 4.1. Seed Assembly and Extract Preparation

**Figure 9:**
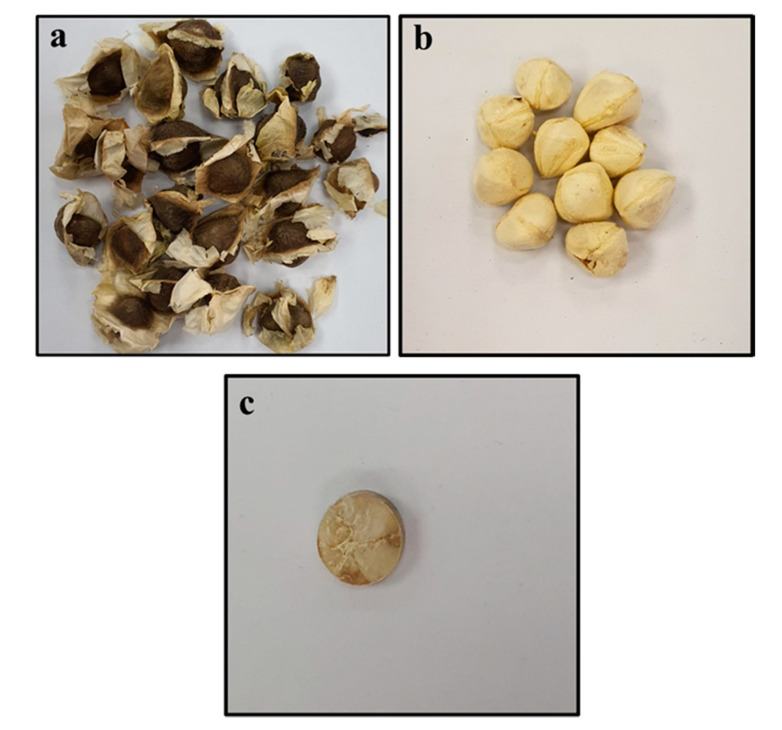
Pictorial view of the pelletization of *Moringa oleifera* seed samples, showing: (**a**) as-purchased *Moringa oleifera* seeds, (**b**) *Moringa oleifera* seeds without coat, (**c**) pelletized seed sample.

All the extracts used in this study were prepared from good quality MOSs procured from a place where they are naturalized, Dammam in Saudi Arabia (originally imported from India). The seeds, without a shell, were crushed to obtain fine powder, followed by compression to get pellets. Figure 9a–c shows different steps of sample production for detailed analysis with the LIBS technique. Diversified ratios of the MOS powder between 5–25 g was mingled with 220 mL of ethanol and were stirred for 5 h, then the MOS solutions were filtered and put into a rotary evaporator to get a dry powder. The samples (MOSs) were subjected to GC-MS and anticancer activity and antibacterial activity tests.

### 4.2. LIBS Setup

**Figure 10:**
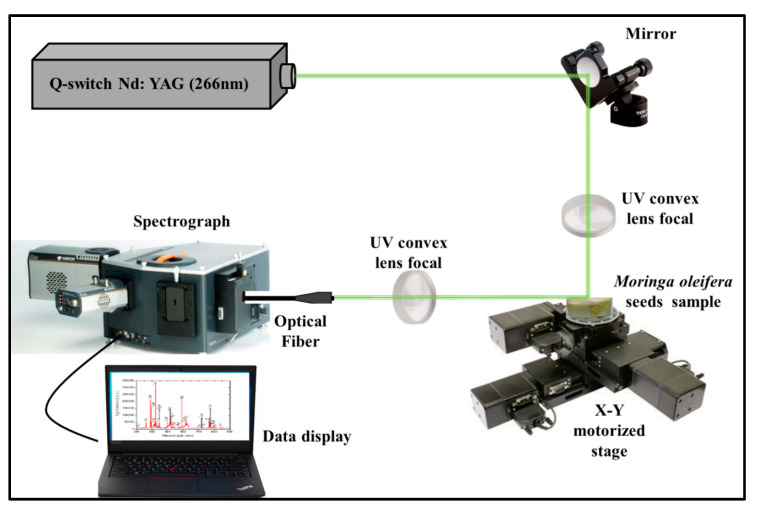
Schematic illustration of the LIBSsetup employed for the detection of vital elements present in MOS.

Figure 10 depicts the modified LIBS setup that was employed to determine the chemical constituents (elemental compositions) present in the MOS specimens. A quadrupled Q-switched Nd:YAG pulse laser (QUV-266-5) with an energy of 30 mJ, a pulse width of 8 ns, a 266 nm wavelength and a 20 Hz repetition rate was used with a UV convex lens of focal length 30 mm to focus the pulses onto the MOS pellets (which acted as the target) to ablate them. A plasma plume was generated when the target was ablated by the laser source. The emitted plasma was detected/collected using a fiber optic system positioned at 45° and the other end (500 mm) was connected to a spectrometer (Andor SR 500i-A) via grating with approximately 1200 lines per mm. An automated sample holder able to move across the plane was used to mount the pellet to avoid the formation of crusts on the surface of the target as a result of several laser pulses on the sample. The spectrum was recorded by an Intensified charge-coupled device (ICCD) camera (Model iStar 320T, 690 × 255 pixels with delay time setting at 300 ns) and data were transferred to an interfaced online computer (PC).

### 4.3. GC-MS Measurements

GC-MS (Shimadzu GC-2010 Plus) was applied for the analysis of ethanol MOS extracts that were provided by a split/splitless auto-injector (AOC-20i series) and coupled to a QP2010 Ultra single quadrupole instrument. The secession with GC was accomplished by using an Rxi-5MS fused silica capillary column (Restek, USA) with 30 m long, 0.25 mm wide and 100 µm thick films. The temperature was raised at a constant rate (5 °C/min) from the initial 60 °C (retained for 0.5 min) up to a final 280 °C (kept for 5 min). The temperature of the inlet was 270 °C (in the splitless mode) and helium (used as carrier gas of purity 99.99%) was blown through the MS transfer line (280 °C) at a 1 mL/min rate. The electron impact modes were used to operate the ion source (70 eV of energy at 250°C) and the mass spectrum (full scan) was recorded in the range of 33–550 *m*/*z*. The spectrum values were gained by regulating the GC-MS and via a GC-MS solution. The compounds of emanated volatiles were disclosed by means of the NIST-11 and WILEY-9 libraries and by calculating the relative area of each compound.

### 4.4. Anticancer Activity of MOS Extract

#### 4.4.1. In Vitro Cell Viability and Cell Culture Assay

As already mentioned, the anticancer activities of the MOS extract were evaluated by treating HCT-116 and normal (HEK-293) human cell lines. The cells were obtained from Dr. Khaldoon M. Alsamman, College of Applied Medical Science, Imam Abdulrahman Bin Faisal University, Dammam, Saudi Arabia. First, cells were cultured in 96 well-plates according to the earlier specified method. Dulbecco’s modified Eagle’s medium (DMEM), supplemented with other reagents (selenium chloride, fetal bovine serum, L-glutamine and antibiotics), was added to the plates for cell growth [29,30].

The cells were treated with varying concentrations of MOS extracts (30–100 µg/mL) for 48 h, whereas in the control, no MOS extract was added. Equal concentrations of a solvent, dimethyl sulfoxide (DMSO) were used in both the control and MOS-treated samples. After 48 h, an MTT assay was performed for 4 h (Molecules, Wellington, New Zealand). Afterward, the growth medium was removed from the plates and (DMSO) was added to every well so the MTT formed formazan crystal. Then, the wells containing the cell cultures were checked at a wavelength of 570 nm via a micro-plate reader (Bio-Rad Labs., Boston, MA, USA). Finally, the recorded readings were analyzed through inbuilt software (Version 5.0, GraphPad Prism) and the statistical significance was studied via ANOVA tests.

#### 4.4.2. Nuclear Staining via DAPI

The cells were treated with MOS extract (at 0.066 µg/mL) for 48 h and, in the control group, no MOS extract was added. The effects of MOS extract on cell nuclei were estimated with DAPI staining. Cold paraformaldehyde (4%) was used to pretreat the cells, which were then washed with 0.1% of Triton X-100 made from phosphate-buffered saline (PBS). At the same time, both the control and MOS- treated cells were stained using PBS with DAPI of 1.0 μg/mL concentration followed by rinsing with X-100 (0.1%). Cell morphologies of both cells (control and the treated) were analyzed *via* (CSM) (CSM-Zeiss, Frankfurt, Germany).

### 4.5. Antimicrobial Activity Assessment of MOS Extract

The effect of MOS extract on antibacterial efficacy at 50, 100, 150, 200 and 250 µg/mL against the *E. coli* ATCC35218 and *S. aureus* ATCC29213 strains was assessed using the AWD method. The inoculum was made from fresh bacteria grown overnight at 37 °C using nutrient broth (NB). This was followed by the preparation of inoculum to 0.5 McFarland standard and 100 µL of inoculum were disseminated over the surface of Mueller–Hinton agar (MHA) plates and dried in an aseptic environment. Next, the inoculated plates (6 mm) were punched using a sterile cork-borer. From the prepared extract, around 50 μL were added to the wells and further kept at 37 °C for an overnight incubation. Thereafter, the bacterial inhibition zone diameter over the entire well was recorded to evaluate the inhibition by the MOS extracts [29].

### 4.6. Antimicrobial Activity Assessment of MOS Extract Using SEM

The effect of MOS extract was additionally studied to investigate the structural damage caused to the selected bacteria by using SEM. The adjusted bacterial cell density, as described above, was treated with 250 µg/mL MOS for overnight incubation. The assay was carried out as per the protocol described by Rehman et.al [29].

### 4.7. Statistical Analysis

In the present study, cell viability data are presented as mean (±) standard deviation (SD), which were obtained from three independent experimental repeats. One-way ANOVA followed by Dunnett’s post hoc test with GraphPad Prism software version 5.0 (GraphPad Software, Inc., La Jolla, CA, USA) was done for the statistical analysis. *p* < 0.05 was considered to indicate a statistically significant difference.

## 5. Conclusions

The LIBS and GC-MS techniques were used to identify and quantify the elemental compositions of MOS extract. The antibacterial and antiproliferative effectiveness of the MOS extracts was evaluated. The LIBS spectra revealed the presence of various nutritional elements in the MOSs that are important for health. The GC-MS analysis reconfirmed the presence of several bioactive compounds in the MOS extract. The MTT assay and DAPI staining showed a significant impact of the MOS extract on the inhibition of the growth of the HCT-116 cells and the insignificant inhibitory action of the extract on the HEK-293 cells, indicating the excellent specificity of the extracts towards the cancer cells. The MOS extracts showed strong antibacterial activity in terms of the growth inhibition and morphogenic changes against *S. aureus* compared to *E. coli,* owing to their cell wall differences. Therefore, it is established that MOS extract can be a prospective antibacterial and anticancer agent for functional pharmaceutical formulations.

## Figures and Tables

**Table 1:** The detection of spectral lines of the different elements present in *MOS* by using our LIBS system.

Name of Element	Wavelength (nm)	Transition Configuration	LIBS Signal Intensity (arbitrary unit(a.u.))
Ca	422.6	3*p*^6^ *4s*^2 1^S_0_→3*p*^6^ *4s 4p* ^1^P°_1_	4960.9
	518.8	3*p*^6^ *4s* 4*p* ^1^P°_1_→3*p*^6^ *4s 5d* ^1^D_2_	2967.1
	527.0	3*p*^6^ *3d 4s* ^3^D_3_→>3*p*^6^ *3d 4p* ^3^P°_2_	3196.3
Fe	248.3	3*d*^6^ *4s*^2 5^D_4_→3*d*^6^ *(^5^D) 4s 4p* (^1^P°) ^5^F°_5_	2114.1
	252.2	3*d*^6^ *4s^2 5^D_4_*→3*d*^6^ *(^5^D) 4s 4p* (^1^P°) ^5^D°_4_	1810.1
	259. 9	3*d*^6^ *(^5^D) 4s* ^6^D_9/2_→3*d*^6^ *(^5^D) 4p* ^6^D°_9/2_	2061.9
K	414.9	*3p^5^ 3d*^3^P°_0_→3*p*^5^ 4*p* ^3^D_1_	2347.6
	430.5	*3p^5^ 3d*^3^P°_2_→3*p*^5^ 4*p* ^1^D_2_	2270.9
	532.3	*3p^6^ 4p*^2^P°_1/2_→3*p*^6^ 8*s* ^2^S_1/2_	2639.7
Mg	518.3	3*s 3p* ^3^P°_2_→*3s 4s* ^3^S_1_	1753.5
Mn	259.3	3*d*^5^ *(^6^S) 4s* ^7^S_3_→3*d*^5^ *(^6^S) 4p* (^7^P°_3_)	1843.9
Na	249.3	2*s*^2^ 2*p^5^ 3s* ^1^P°_1_→2*s*^2^ 2*p^5^ 3p* ^1^S_0_	2128.4
	251.5	2*s*^2^ 2*p^5^ 3p* ^3^S_1_→2*s*^2^ 2*p^5^ (*^2^P°_1/2_*) 3d* ^2^[→]°_2_	1834.7
P	253.5	3*s*^2^ *3p^3^* ^2^P°_3/2_→3*s*^2^ *3p^2^ (^3^P) 4s* ^2^P_3/2_	2100.0
	255.3	3*s*^2^ *3p^3^* ^2^P°_1/2_→3*s*^2^ *3p^2^ (^3^P) 4s* ^2^P_1/2_	2032.1
S	416.2	3*s*^2^ *3p^2^ (^3^P) 4p* ^4^D°_7/2_→3*s*^2^ *3p^2^ (^3^P) 4d* ^4^F_9/2_	1987.8
Se	418.09	4*s*^2^ *4p*^2^ (^3^P) *5p* ^4^D°_7/2_→4*s*^2^ *4p*^2^ (^3^P) *5d* ^4^F_9/2_	1969.7
	522.7	4*s*^2^ *4p*^2^ (^3^P) *5s* ^4^P_5/2_→4*s*^2^ *4p*^2^ (^3^P) *5p* ^4^D°_7/2_	2002.0
Zn	250.1	3*d*^10^ *4p* ^2^P°_1/2_→3*d*^10^ *5s* ^2^S_1/2_	1751.4
	255.7	3*d*^10^ *4p* ^2^P°_3/2_→3*d*^10^ *5s* ^2^S_1/2_	1762.3
C	426.7	2*s*^2^ 3*d* ^2^D_5/2_→2*s*^2^ 4*f* ^2^F°_7/2_	2379.0
	247.8	2*s*^2^ *2p^2^* ^1^S_0_→2*s*^2^ *2p 3s* ^1^P°_1_	1831.6
O	418.9	2*s*^2^ *2p*^2^ (^1^D) *3p* ^2^F°_7/2_→2*s*^2^ *2p*^2^ (^1^D) *3d* ^2^G_9/2_	2003.4

**Table 2:** Volatile compounds identified in MOS using GC-MS analysis.

No.	Compounds	RT	Peak area (%)
	**Esters**		
1	Propanoic acid, 2-oxo-, methyl ester	6.293	0.76
2	Acetic acid, ethoxyhydroxy-, ethyl ester	7.043	0.04
3	Diethoxymethyl acetate	12.827	0.09
4	2-Propenoic acid, 2-methyl-, 2-hydroxypropyl ester	13.134	0.02
5	6,9,12-Octadecatrienoic acid, phenylmethyl ester, (*Z*,*Z*,*Z*)-	17.797	0.01
6	Cyclopentanecarboxylic acid, 4-tridecyl ester	19.179	0.03
7	1-Cyclohexene-1-carboxylic acid, 2,6,6-trimethyl-, methyl ester	20.058	0.13
8	Hexanoic acid, 4-hexadecyl ester	28.291	0.07
9	Phthalic acid, diethyl ester	29.568	0.14
10	2-Propenoic acid, pentadecyl ester	31.625	0.59
11	Acetic acid, 3,7,11,15-tetramethyl-hexadecyl ester	33.627	0.02
12	Phthalic acid, dibutyl ester	35.692	0.07
13	Phthalic acid, diisobutyl ester	35.760	0.15
14	Palmitoleic acid, methyl ester	36.285	0.03
15	Pentadecanoic acid, 13-methyl-, methyl ester	36.642	0.09
16	9-Hexadecenoic acid, ethyl ester	37.652	0.15
17	Hexadecanoic acid, ethyl ester (Ethyl palmitate)	37.993	0.78
18	Heptadecanoic acid, ethyl ester	38.416	0.46
19	Propanoic acid, 3-mercapto-, dodecyl ester	38.588	0.25
20	9-Octadecenoic acid, methyl ester, (*E*)- (Methyl elaidate)	40.159	1.52
21	Oleic acid, methyl ester (Methyl oleate)	40.255	0.15
22	l-(+)-Ascorbic acid 2,6-dihexadecanoate	40.561	0.10
23	Ethyl oleate	41.389	6.03
24	Octadecanoic acid, ethyl ester (Ethyl stearate)	41.774	0.53
25	9-octadecenyl ester (Oleyl oleate)	43.516	0.34
26	2,3-dihydroxypropyl elaidate	43.794	13.48
27	9-Octadecenoic acid, 1,2,3-propanetriyl ester	44.287	0.90
28	Docosanoic acid, ethyl ester	45.273	0.37
29	Oleoyl chloride	46.066	0.65
30	9-Octadecenoic acid (*Z*)-, 2,3-dihydroxypropyl ester	47.099	11.35
31	Glycidol stearate	47.522	1.76
32	Hexadecanoic acid, 2-hydroxy-1-(hydroxymethyl)ethyl ester	47.803	1.57
	**Alcohols**		
33	1,4-Cyclohexanediol, trans-	7.224	0.02
34	1,2-Propanediol, 3-methoxy-	7.430	0.05
35	Ethanol, 2,2-diethoxy-	7.535	0.31
36	1,2,4-Butanetriol	9.496	0.04
37	Glycerin	11.180	1.35
38	1-Butanol, 4-(ethylthio)-	11.742	0.02
39	Methoxyacetaldehyde diethyl acetal	11.883	0.02
40	1-Dodecanol	18.431	0.48
41	1-Tetradecanol	24.152	0.42
42	1,3-Propanediol, 2-ethyl-2-(hydroxymethyl)-	25.444	5.52
43	1-Tridecanol	26.331	1.06
44	3-Hexadecanol	31.348	0.05
45	3-Heptadecanol	35.561	0.06
	**Aldehydes**		
46	Butanal, 3-hydroxy-	8.375	0.00
47	Heptanal	10.006	0.02
48	Octanal	12.567	0.04
49	Nonanal	15.790	0.09
50	2-Methyl-oct-2-enedial	20.611	0.04
51	Undecanal	23.521	0.02
52	Tridecanal	34.455	0.01
53	10-Octadecenal	37.418	0.09
54	9-Octadecenamide	37.551	0.09
55	cis-9-Hexadecenal	38.241	0.55
56	cis-13-Octadecenal	47.228	1.92
	**Ketones**		
57	2-Propanone, 1,1-dimethoxy-	6.841	3.86
58	2-Butanone	8.151	0.03
59	Dihydroxyacetone	8.969	0.14
60	1,2-Cyclopentanedione	10.172	0.14
61	2-Propanone, 1-(1,3-dioxolan-2-yl)-	10.867	0.04
62	1,3-Dioxol-2-one,4,5-dimethyl-	13.528	0.28
63	2-Heptanol, 5-ethyl-	14.306	0.01
64	2-Methyl-4-octanone	16.466	0.03
65	2-Pentanone, 3,4-epoxy-	18.925	0.03
66	1-Oxa-spiro[4.5]deca-6,9-diene-2,8-dione	37.006	0.05
67	*Z*-11-Pentadecenol	39.209	0.05
68	Cyclopentadecanone, 2-hydroxy-	39.822	0.13
69	Cyclopentadecanone	43.430	0.21
70	2-Tetradecanone	46.691	0.34
	**Acids**		
71	Acetic acid, (acetyloxy)-	5.884	0.18
72	Butanoic acid, 3-hydroxy-	10.594	0.03
73	Octanoic acid	17.421	0.03
74	Nonanoic acid	20.363	0.02
75	n-Hexadecanoic acid (Palmitic acid)	37.305	0.05
76	Oleic acid	40.943	22.53
	**Furans and lactones**		
77	Furfural	7.624	0.02
78	2(5H)-Furanone	9.840	0.04
79	2-Hydroxy-gamma-butyrolactone	12.081	0.23
80	2,5-Dimethyl-4-hydroxy-3(2H)-furanone	14.099	0.19
81	1,2-Ethanediol, 1-(2-furanyl)	19.096	0.03
82	5-Hydroxymethylfurfural	19.445	0.36
83	3-Deoxy-d-mannoic lactone	28.924	0.52
	**Nitrogen-containing compounds**		
84	*N*,*N*-Dimethylaminoethanol	5.216	0.12
85	1,3,5-Triazine-2,4,6-triamine	14.778	0.68
86	Acetic acid, 2-(*N*-methyl-*N*-phosphonatomethyl)amino-	15.617	0.11
87	1-Heptadecanamine	18.667	0.07
88	Nonanamide	23.034	0.02
89	Dodecanamide (Lauryl amide)	33.331	0.04
90	Tetradecanamide (Myristic amide)	37.717	0.44
91	Hexadecanamide (Palmitic amide)	41.718	1.26
92	Docosenamide	44.987	6.04
93	Nonadecanamide	45.352	0.32
	**Sulfur-containing compounds**		
94	Sulfurous acid, cyclohexylmethyl hexadecyl ester	23.173	0.67
	**Hydrocarbons**		
95	1-Butene, 4,4-diethoxy-2-methyl-	9.425	0.07
96	1-Methyl-2-octylcyclopropane	12.171	0.07
97	2-Trifluoroacetoxytridecane	13.923	0.03
98	trans-2,3-Epoxynonane	23.642	0.03
99	2-Heptafluorobutyroxypentadecane	23.887	0.04
100	1,2-Epoxyundecane	24.016	0.02
101	Heptacosane	24.357	0.03
102	1-Heptadecene	29.262	0.33
103	Octadecane, 1,1’-[(1-methyl-1,2-ethanediyl)bis(oxy)]bis-	29.426	0.01
104	1-Nonadecene	33.847	0.22
	**Pyrans**		
105	Tetrahydro-4H-pyran-4-ol	17.074	0.03
106	4H-Pyran-4-one, 2,3-dihydro-3,5-dihydroxy-6-methyl-	17.198	0.02
	**Others**		
107	5,6-Dihydroxypiperazine-2,3-dione dioxime	8.320	0.02
108	Tetraethyl silicate	11.807	0.04
109	Silanediol, dimethyl-, diacetate	17.300	0.07
110	D-Allose	26.609	0.19
111	Phenol, 2,4-bis(1,1-dimethylethyl)-	27.430	0.51
112	Oxirane, hexadecyl-	34.898	0.02
113	Ethyl iso-allocholate	36.936	0.06
114	Oleic anhydride	43.700	3.96

RT: Retention time in minutes.
